# Reorganization of corticospinal tract fibers after spinal cord injury in adult macaques

**DOI:** 10.1038/srep11986

**Published:** 2015-07-01

**Authors:** Hiroshi Nakagawa, Taihei Ninomiya, Toshihide Yamashita, Masahiko Takada

**Affiliations:** 1 Systems Neuroscience Section, Primate Research Institute, Kyoto University, Inuyama, Aichi 484-8506, Japan; 2 Department of Molecular Neuroscience, Graduate School of Medicine, Osaka University, Suita, Osaka 565-0871, Japan

## Abstract

Previous studies have shown that sprouting of corticospinal tract (CST) fibers after spinal cord injury (SCI) contributes to recovery of motor functions. However, the neuroanatomical mechanism underlying the functional recovery through sprouting CST fibers remains unclear. Here we investigated the pattern of reorganization of CST fibers below the lesion site after SCI in adult macaques. Unilateral lesions were made at the level between the C7 and the C8 segment. The extent of spontaneous recovery of manual dexterity was assessed with a reaching/grasping task. The impaired dexterous manual movements were gradually recovered after SCI. When anterograde tract tracing with biotinylated dextran amine was performed to identify the intraspinal reinnervation of sprouting CST fibers, it was found that the laminar distribution of CST fibers was changed. The sprouting CST fibers extended preferentially into lamia IX where the spinal motor neuron pool was located, to innervate the motor neurons directly. Instead, few, if any, CST fibers were distributed in the dorsal laminae. The present results indicate that CST fibers below the lesion site after SCI in macaques are reorganized in conjunction with the recovery of dexterous manual movements.

Dexterous manual movements strongly relate to the development of the corticospinal tract (CST) in mammals and, therefore, become severely impaired after spinal cord injury (SCI)^1^. Although the adult mammalian spinal cord fails to regenerate once lesioned, motor impairments are often followed by spontaneous recovery. Indeed, previous studies have shown that after unilateral SCI in primates, at least part of CST fibers extend beyond the lesion site with restoration of manual dexterity^2^,^3^,^4^,^5^. It has also been reported in rodents that sprouting CST fibers after SCI extend frequently into the medial gray matter where spinal interneurons are located, given that only a few number of CST fibers normally connect with spinal motor neurons directly^6^,^7^,^8^. In this context, the pattern of intraspinal reinnervation of sprouting CST fibers could differ in rodents and primates. However, it still remains to be elucidated where in the spinal cord (which spinal laminae) the sprouting CST fibers after SCI extend in primates, though primate CST fibers are known to normally display a widespread distribution over all spinal laminae^9^,^10^,^11^.

To explore the neuroanatomical mechanism that underlies the functional recovery after SCI in primates, adult macaques underwent spinal cord lesions at the cervical cord level in the present study. Employing this primate SCI model, we provide evidence that the sprouting CST fibers are reorganized in conjunction with the restoration of dexterous manual movements.

## Results

### Extent of spinal cord lesions

**Figure 1:**
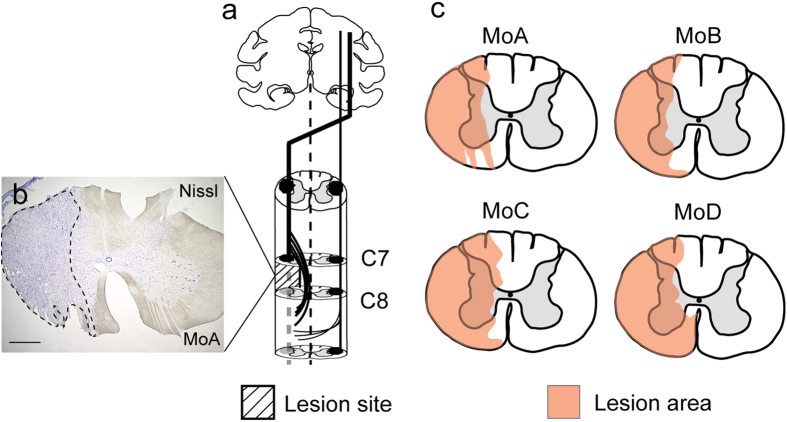
Primate SCI model and extent of spinal cord lesions. (**a**) Unilateral SCI made at the level between the C7 and the C8 segment. (**b**) Extent of the lesion area (broken lines; Nissl staining) in MoA. Scale bar: 1 mm. (**c**) Extent of SCI (in pink) in a representative transverse section in MoA–MoD. Note that, in all cases, dorsolaterally-situated CST fibers are fully removed, and part of the gray matter in the medial aspect is left intact to retain a route of sprouting CST fibers.

In our SCI model in which a border region between the C7 and the C8 segment was lesioned (Fig. 1a), part of the gray matter in the medial aspect was left intact to retain a route of sprouting CST fibers. The extent of spinal cord lesions in a transverse section was 61.1%, 55.5%, 71.3%, and 71.5% of the total area on the lesioned side in MoA–MoD, respectively (Fig. 1b,c). In each case, dorsolaterally-situated CST fibers were fully removed (Fig. 1b,c). The spinal cord lesions in MoB and MoC infringed, to some extent, upon the C7 segment *per se*. Results of anterograde tract tracing with biotinylated dextran amine (BDA) indicated that CST fibers travelling through the dorsolateral funiculus were not observed at all below the lesion site in any of the four monkeys (see Fig. 3k). Ventrally-situated CST fibers through other descending tracts appeared to be preserved in the three monkeys except MoD (Fig. 1c). The rubrospinal tract region was largely damaged in all cases, and the reticulospinal tract region was damaged partly in MoA–MoC and completely in MoD (Fig. 1b,c).

### Behavioral analyses

**Figure 2:**
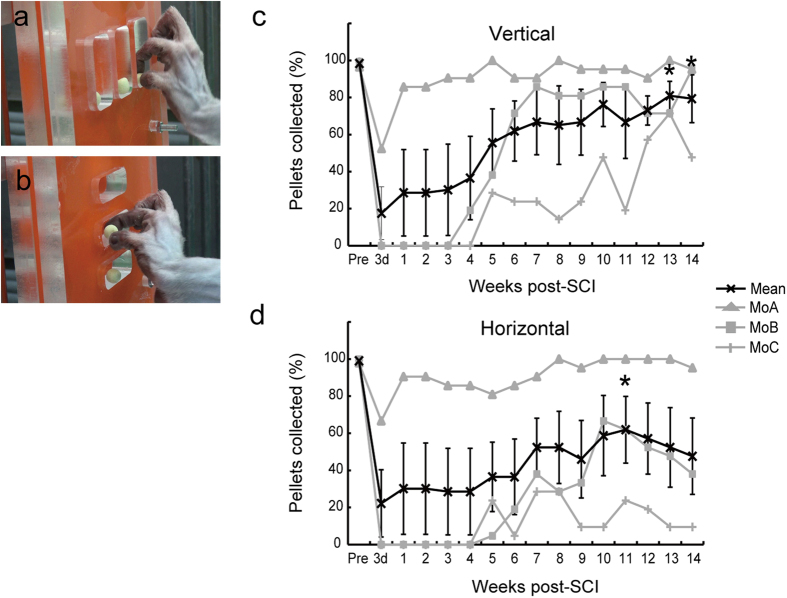
Behavioral analyses of dexterous manual movements. (**a**,**b**) Reaching/grasping task. The monkey is required to reach for a vertical (**a**) or horizontal (**b**) slot, grasp a pellet, and convey it to the mouth within 10 seconds. (**c**,**d**) Changes in the ratios of pellets collected successfully in the vertical-type (**c**) and horizontal-type (**d**) tasks in a daily test (see the text for details). 3d, 3 days post-SCI.

To assess the extent to which dexterous manual movements were recovered spontaneously in our SCI model, a reaching/grasping task was performed (Fig. 2a,b). Behavioral analyses were started 3 days after SCI and continued once a week until 14 weeks post-SCI.

In the reaching/grasping task, the ratios of pellets collected successfully in vertical and horizontal types of grasping movements were decreased quickly after SCI. Then, dexterous manual movements were gradually restored, although there were certain individual differences in the process of functional recovery, probably due to the extent of the spinal cord lesions (Fig. 2c,d).

### Histological analyses

**Figure 3:**
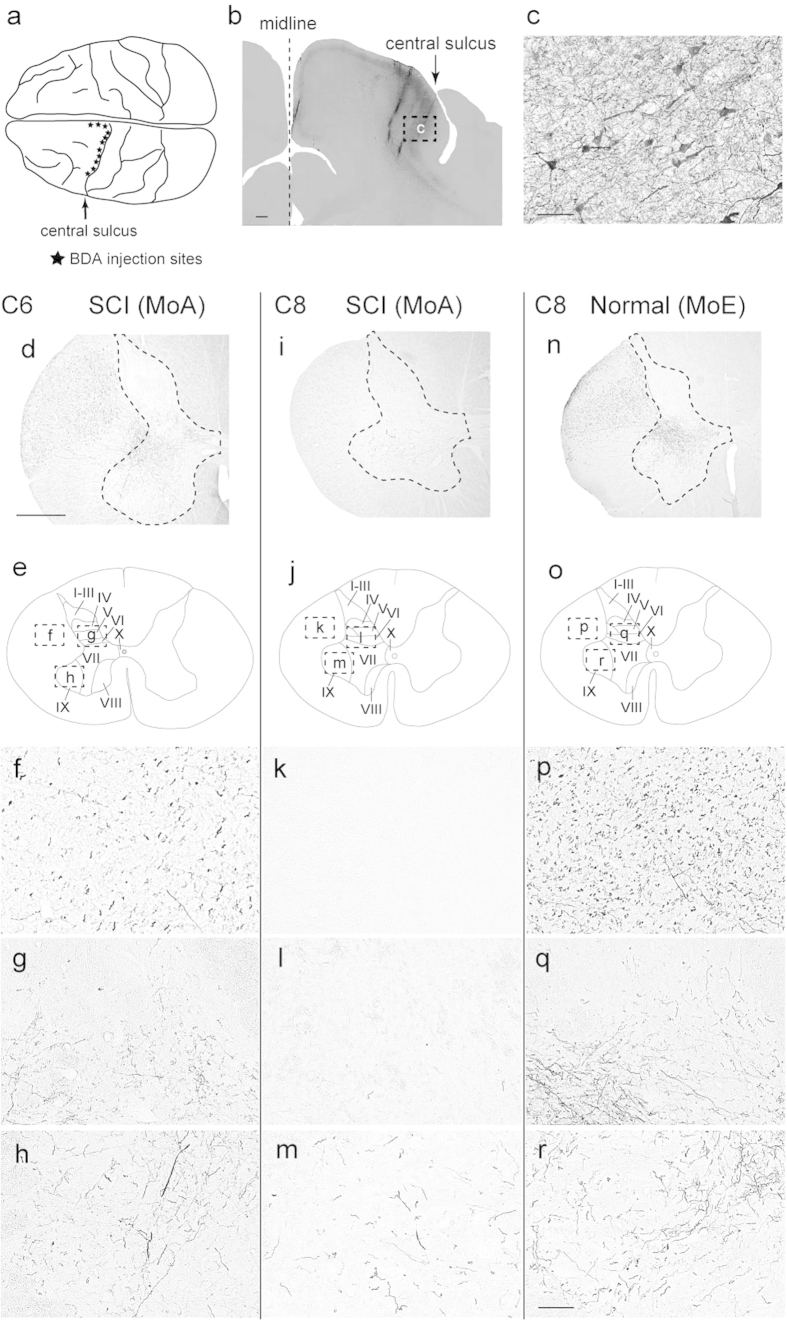
Immunohistochemical analyses of the sites of BDA injections in the MI and the distribution of BDA-labeled CST fibers. (**a**) Sites of BDA injections in MI regions representing the forelimb, trunk, and hindlimb (denoted with stars). (**b**) Example of the injection site in the forelimb region. Scale bar: 1 mm. (**c**) Higher-power magnification of a rectangular area in b. Scale bar: 50 μm. (**d**,**i**,**n**) Representative transverse sections through the C6 (**d**) and C8 (**i**) segments in an SCI model (MoA) and the C8 segment (**n**) in a normal control (MoE). Scale bar: 1** **mm. (**f**–**h**) Higher-power magnification of rectangular areas in **e**. (**k**–**m**) Higher-power magnification of rectangular areas in j. (**p**–**r**) Higher-power magnification of rectangular areas in o. **f**,**k**,**p**: Laterally-situated CST fibers. g,l,q: Laminae IV to VI. **h**,**m**,**r**: Lamina XI. Scale bar: 100 μm.

**Figure 4:**
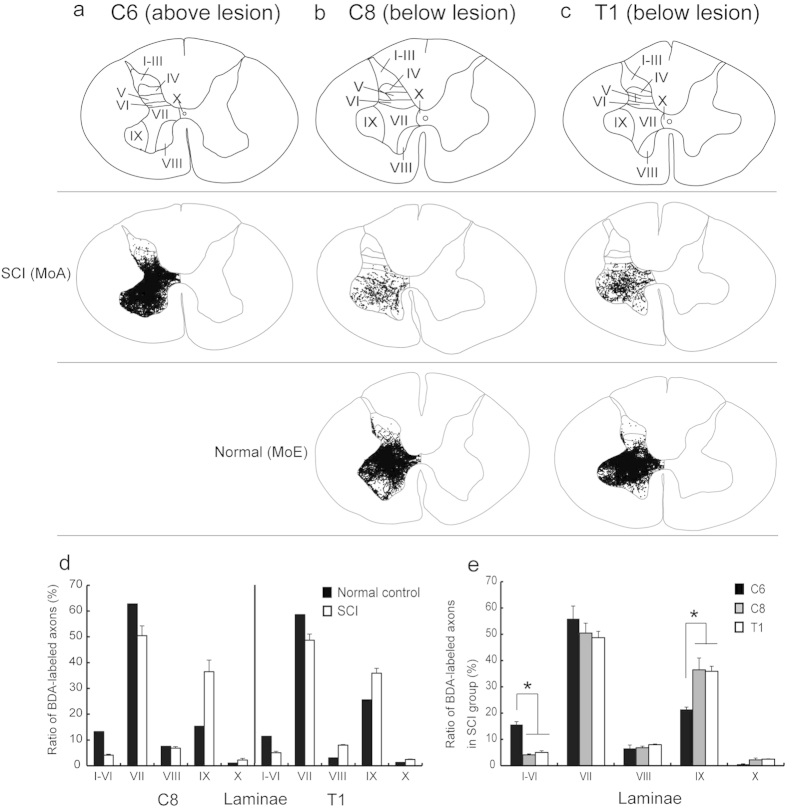
Patterns of laminar distribution of anterogradely-labeled CST fibers in an SCI model (MoA) and a normal control (MoE). (**a**) Distribution of CST fibers in the C6 segment above the lesion site (intact control) in MoA. (**b**,**c**) Distributions of CST fibers in the C8 (**b**) and T1 (**c**) segments below the lesion site in MoA (upper row) and MoE (lower row). In each segment, the laminar parcellation is depicted in a representative transverse section. (**d**) Histograms showing the ratio of BDA-labeled axons in each lamina to the total in the C8 and T1 segments in the SCI group and normal control. (**e**) Histograms showing the ratio of BDA-labeled axons in the C6, C8 and T1 segments in the SCI group. Error bars denote SEM, n = 3. Dunnet test, *P < 0.05.

To investigate the distribution pattern of sprouting CST fibers after SCI at the level between the C7 and the C8 segments, data obtained from anterograde tract tracing of CST fibers and terminal button-like swellings were analyzed in the C8 and T1 segments (below the lesion site), in comparison with the intact C6 segment and the normal C8 and T1 segments. In these control cases, anterogradely-labeled CST fibers after BDA injections into the contralateral primary motor cortex (MI; Fig. 3a–c) were distributed extensively over dorsal laminae, though they were located primarily in lamina VII and then in lamina IX (Figs 3d–h,n–r and 4a–e). A much smaller number of BDA-labeled CST fibers were found in both of the C8 and T1 segments in the SCI group than in the control cases (C6: C8, *P *= 0.038; C6: T1, *P *= 0.04; Figs 3d–r and 4a–e). Consistent with the distribution in the control cases, about half of the labeled CST fibers in these segments were distributed in lamina VII. In remarkable contrast with the control cases, the ratio of CST fiber labels in lamina IX after SCI went up to 36.5 ± 4.5 (C8) and 36.0 ± 0.2 (T1) of the total (C6, 21.4 ± 0.9). However, those in dorsal laminae were largely reduced to a few percent of the total (C6: C8, *P *= 0.028; C6: T1, *P *= 0.032; Fig. 4a–e). The values for laminae I–VI in the C6, C8, and T1 segments were 15.6 ± 1.1, 4.1 ± 0.4 and 5.0 ± 0.6, respectively.

**Figure 5:**
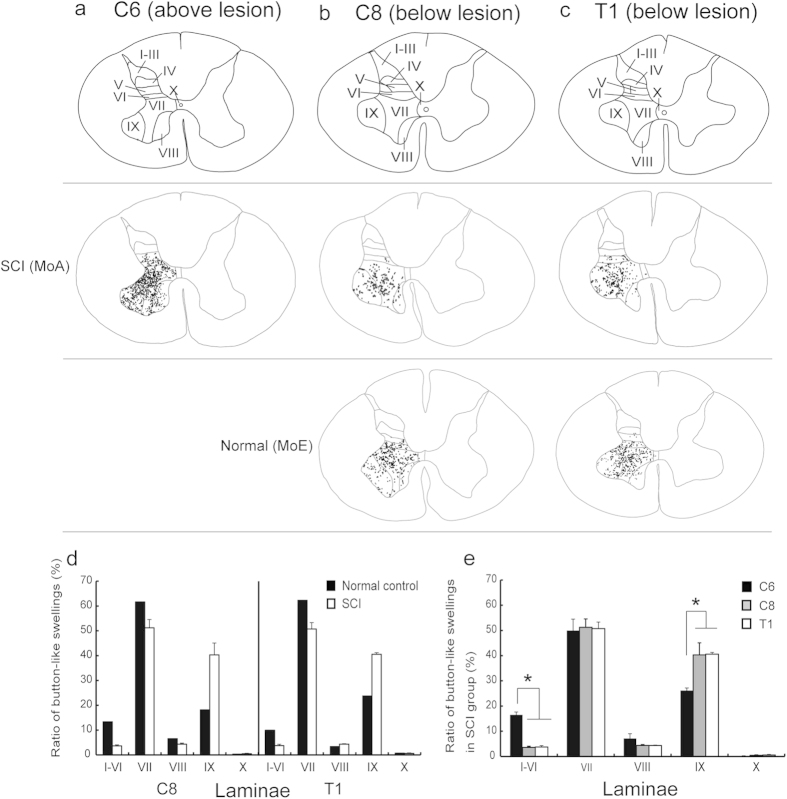
Patterns of laminar distribution of button-like swellings of anterogradely- labeled CST fibers in an SCI model (MoA) and a normal control (MoE). (**a**) Distribution of button-like swellings in the C6 segment above the lesion site (intact control) in MoA. (**b**,**c**) Distributions of button-like swellings in the C8 (**b**) and T1 (**c**) segments below the lesion site in MoA (upper row) and MoE (lower row). In each segment, the laminar parcellation is depicted in a representative transverse section. (**d**) Histograms showing the ratio of BDA-labeled button-like swellings in each lamina to the total in the C8 and T1 segments in the SCI group and normal control. (**e**) Histograms showing the ratio of BDA-labeled button-like swellings in the C6, C8 and T1 segments in the SCI group. Error bars denote SEM, n = 3. Dunnet test, *P < 0.05.

Essentially the same results were obtained concerning the distribution change in button-like swellings after SCI. In the intact C6 segment and the normal C8 and T1 segments, button-like swellings labeled from the contralateral MI were observed widely over dorsal laminae, though their dense accumulations were seen predominantly in lamina VII and, to a lesser extent, in lamina IX (Fig. 5a–e). The labeled button-like swellings were much fewer in both of the C8 and T1 segments after SCI than in the control cases (C6: C8, *P *= 0.028; C6: T1, *P *= 0.02). In these segments, the ratio of button-like swelling labels in lamina IX went up to 40.3 ± 4.7 (C8) and 40.5 ± 0.7 (T1) of the total (C6, 26.2 ± 1.0). However, those in dorsal laminae were greatly reduced to almost none (C6: C8, *P *= 0.038; C6: T1, *P *= 0.04; Fig. 5a–e). The values for laminae I–VI in the C6, C8, and T1 segments were 15.6 ± 1.1, 4.1 ± 0.4 and 5.0 ± 0.6, respectively.

## Discussion

The purpose of our work is to elucidate the pattern of reorganization of CST fibers in the spinal cord after SCI in macaques. We have defined evidence that sprouting CST fibers in a primate SCI model are reorganized in conjunction with the recovery of dexterous manual movements. Although it has been considered that axonal sprouting is crucial to spontaneous recovery of motor functions after SCI^12^, the neuroanatomical mechanism underlying the functional recovery still remains to be known in primates. Previous studies have shown that CST fibers extend across the lesion site with functional recovery after SCI in adult monkeys^2^,^3^,^4^,^5^. This indicates that the mature primate brain has a capacity of axonal regrowth, and thus, sprouting CST fibers may contribute directly to spontaneous recovery of dexterous manual movements. To restore manual dexterity after SCI, sprouting CST fibers are required to transmit cortically-derived motor commands to spinal motor neurons, which is likely to be mediated through direct and indirect pathways that constitute the CST. There is a consensus that CST fibers in primates project mainly to laminae V–VII (through the indirect pathway via spinal interneurons) and lamina IX (through the direct pathway) (present data; see also refs 8, 9, 10). According to recent studies^13^,^14^, the spinal interneurons may mediate motor commands necessary for hand muscle activity to the motor neurons in intact monkeys. Therefore, it is of general interest to determine which spinal laminae the sprouting CST fibers after SCI may extend to promote the functional recovery in primates. In our study, a series of experiments was designed to address this issue.

First, we analyzed the process of spontaneous recovery of manual dexterity in our primate SCI model. To assess the extent of behavioral recovery, a reaching/grasping task was employed in the present experiments. Our behavioral analyses demonstrated that impaired dexterous manual movements were gradually recovered after SCI. Since the extent of SCI in MoB and MoC infringed, at least partly, upon the C7 segment *per se*, dexterous manual movements were affected more severely and recovered more slowly in these monkeys than in MoA with a restricted lesion at the border between the C7 and the C8 segments. It should also be described that the impaired grasping movements of the horizontal type were not so easily restored as the vertical type, probably because the horizontal type movements required finer wrist control than the vertical type. In human patients, it was reported that motor functions were recovered with an increase in muscle activity after cervical cord injury^15^,^16^.

Next, we applied anterograde tract tracing to elucidate the mode of intraspinal reinnervation of sprouting CST fibers after SCI. As verified in the C6 segment above the lesion site, intact CST fibers are distributed extensively over dorsal laminae in particular abundance in lamina VII. It has been revealed in our study that the sprouting CST fibers extend preferentially into lamina IX where spinal motor neurons are confined, to innervate the motor neurons directly by newly formed button-like swellings. Instead, much fewer CST fibers are directed toward the dorsal laminae. These plastic events may greatly contribute to promote the recovery of dexterous manual movements efficiently and effectively through a limited number of sprouting CST fibers. Schmidlin *et al*.^2^ have reported that movements of the digits elicited by intracortical microstimulation in the contralateral MI in the same SCI model as ours may probably be attributed to such sprouting CST fibers. Of particular interest is that a similar strategy may underlie the formation of compensatory CST projections. It should be noted here that the common neuroanatomical mechanism might also serve to achieve a compensatory spinal innervation by other possible descending fibers, such as ipsilaterally-travelling and recrossing CST fibers^17^,^18^. In addition, previous works have shown that plastic changes in intraspinal circuits through peripheral sensory inputs contribute to functional restoration after SCI^19^,^20^,^21^. It was found in our study that MoA with a milder spinal cord lesion recovered from impaired dexterous manual movements much earlier than MoB and MoC. A similar mechanism at the intraspinal circuit level may also participate in the recovery of motor function in MoA.

The present results clearly indicate that a number of sprouting CST fibers are reorganized below the lesion site in conjunction with the recovery of dexterous manual movements after SCI in primates. This suggests that the mature CST is most likely to have an endogenous mechanism that permits limited but effective axonal sprouting to promote the formation of a direct cortico-motoneuronal pathway. A key factor, for example, a guidance molecule, may be involved in mediating intraspinal reinnervation by sprouting CST fibers. Elucidating a molecular basis for such a plastic event might contribute to the development of effective therapeutic approaches to SCI.

## Materials and Methods

### Animals

Five rhesus monkeys (*Macaca mulatta*) were used for this study: SCI models (MoA–MoD), 4–11 years old, 4.1–5.2 kg; normal control (MoE), 4 years old, 4.9 kg. The monkeys were housed in individual cages in a 12-h light/dark cycle with *ad libitum* access to food and water. The experimental protocol was approved by the Animal Welfare and Animal Care Committee of the Primate Research Institute, Kyoto University. All experiments were conducted in accordance with the Guidelines for Care and Use of Nonhuman Primates (Ver. 3, 2010) issued by the institute.

### Surgical procedures

The monkeys were sedated with a combination of ketamine hydrochloride (10 mg/kg, i.m.) and xylazine hydrochloride (1 mg/kg, i.m.), and then anesthetized with sodium pentobarbital (25 mg/kg, i.v.). Surgical procedures for SCI were performed as previously described^3^,^4^. After the absence in reaction to stimuli (i.e., eyelid flutter and extremity pressure), the skin and axial muscles were dissected at the level of the C3 to T2 segments. Subsequently, laminectomy involving the C5 to T1 segments was performed, the dura mater was cut unilaterally, and a border region between the C7 and the C8 segment was lesioned with a surgical blade (No. 11) and a special needle (27G). In this SCI model, the dorsolateral funiculus was fully injured to remove laterally situated CST fibers, and part of the gray matter was kept intact to retain a route of sprouting CST fibers. After SCI, the dura mater was sutured, a spongel (Astellas, Tokyo, Japan) was placed on it for arrest of hemorrhage, and then the skin and axial muscles were sutured. Finally, the monkeys were given an analgesic (Lepetan; Otsuka, Tokyo, Japan; i.m.; Indomethacin; Isei, Yamagata, Japan; 1 week, oral) and an antibiotic (Viccillin; Meiji Seika, Tokyo, Japan; 40 mg/kg/day for 4 days, i.m.).

### Behavioral task

In each monkey, a behavioral task (reaching/grasping task) was trained for approximately 2 months prior to SCI. After SCI, the task was performed once a week until 14 weeks post-SCI. Behavioral data were analyzed using a video camera. It should also be described here that the lesion site of SCI in MoD was extended partly into the C6 segment, and therefore, the behavioral data for MoD were precluded from the analysis.

Reaching/grasping task. This task was applied to assess a combined skilled behavior of reaching and grasping (i.e., manual dexterity) after SCI at the cervical cord level. A series of movements that monkeys reach for a small piece of food and grasp it was used to assess the recovery of manual dexterity after SCI in primates^22^. The monkey was seated in a primate chair, and an acrylic board (14 cm x 14 cm) with three vertical or horizontal slots was placed in front of the chair. The vertical and horizontal slots were 40 mm long x 22 mm wide x 10 mm deep, and 13 mm long x 40 mm wide x 10 mm deep, respectively, and each slot was filled with a food pellet (diameter, 9 mm; Osaka Maeda Seika, Osaka, Japan). In our reaching/grasping task, each session for the vertical and horizontal tasks consisted of 21 trials (3 trials x 7 times) to pick up a total of 21 pellets, and a trial was considered to be successful when the monkey reached for a slot, grasped a pellet, and conveyed it to the mouth within 10 seconds. On the other hand, a trial was counted as an error when the monkey failed to grasp a pellet or dropped it on the way to the mouth. The baseline value before SCI was represented as the mean of successful numbers (maximum = 21) per session in each of the vertical and horizontal tasks that were performed for 7 days (1 session per day). After SCI, the monkey continuously underwent 1 session per day (daily test). All data were represented as the ratio of the number of successfully collected pellets to the total.

### Anterograde tract tracing of CST fibers

To examine the intraspinal distribution of sprouting CST fibers, anterograde tract tracing with BDA was performed as previously described^10^. Briefly, a 10% solution of BDA (10,000 MW; Invitrogen, Carlsbad, CA, USA) was injected into the MI 7 weeks before sacrifice. Under general anesthesia with sodium pentobarbital (25 mg/kg, i.v.), the monkeys underwent craniotomy to expose the central sulcus. Multiple injections of BDA (150 nl x 127 sites) were made extensively over the forelimb, trunk, and hindlimb regions of the MI through a Hamilton microsyringe (Fig. 2a,b). After the BDA injections, the dural defect was covered with a gelfilm (Pfizer, Kalamazoo, MI, USA), and the craniotomy site was covered with a cranium and was fixed using an adhesive resin.

### Histological procedures

Tissue processing and immunohistochemical procedures were performed as previously described with minor modifications^23^. Under deep anesthesia with an overdose of sodium pentobarbital (50 mg/kg, i.v.), the monkeys underwent perfusion fixation with 10% formalin. Following the perfusion, the spinal cord was post-fixed in the same fresh fixative overnight at 4 °C, immersed in a 30% sucrose solution in 0.1 M phosphate-buffered saline (PBS; pH 7.4) for 2 weeks at 4 °C, and then processed for histological staining for BDA. The spinal cord was cut serially into 40-μm-thick transverse sections, and the brain was cut into 50-μm-thick coronal sections on a freezing microtome. To inhibit endogenous peroxidase, the sections were treated with 0.3% hydrogen peroxide in PBS for 30 min at room temperature. For detection of BDA labeling, the sections were incubated with avidin-biotin-peroxidase complex (ABC Elite; 1:200 dilution; Vector laboratories, Burlingame, CA, USA) in PBS containing 0.1% Triton X-100 overnight at 4 °C. Subsequently, the sections were reacted in 0.05 M Tris-HCl (pH 7.6) containing 0.04% diaminobenzidine tetrahydrochloride (DAB), 0.04% nickel chloride, and 0.006% hydrogen peroxide.

To identify the spinal motor neuron pool in lamina IX, some sections were processed for immunohistochemical staining for choline acetyltransferase (ChAT). The sections were treated with 0.3% hydrogen peroxide in PBS for 30 min, and then incubated with goat anti-ChAT antibody (1:1,000 dilution; Millipore, Temecula, CA, USA) in an incubation medium containing 2% normal donkey serum, 3% bovine serum albumin, and 0.1% Triton X-100 in PBS for 120 min at room temperature, followed by 2 overnights at 4 °C. Subsequently, the sections were incubated with biotinylated donkey anti-goat IgG antibody (1:1,000 dilution; Jackson Immuno Research laboratories, West Grove, PA, USA) in the same fresh incubation medium for 120 min at room temperature, and then with ABC Elite (1:200 dilution) for 90 min at room temperature. Finally, the sections were visualized with the Vector NovaRED substrate kit (Vector laboratories) or the DAB-nickel method (as described above). All sections were mounted onto gelatin coated-glass slides. Anterograde labeling was traced with a Neurolucida software (MicroBrightField, Chiba, Japan).

### Measurements of the extent of spinal cord lesions

The spinal sections were Nissl-stained with 1% Cresyl violet. The extent of spinal cord lesions was measured with an ImageJ software (National Institutes of Health, Bethesda, MD, USA) and expressed as the percentage of the lesioned area to the total area on the lesioned side.

### Quantification of CST fibers and terminal button-like swellings

For histological analysis of sprouting and intact CST fibers, five spinal sections (800 μm apart) through the C6, C8, and T1 segments were used. The numbers of BDA-labeled CST fibers and terminal button-like swellings were counted at x400 magnification in each spinal lamina on the lesioned side, as previously described^11^. Counts of the labeled fibers were done in each spinal lamina even though single fibers travelled across multiple laminae. The details of quantification of anterograde labeling and parcellation of the spinal laminae were as previously described^24^,^25^. Additionally, lamina IX was demarcated through identification of the ChAT-positive (cholinergic) spinal motor neuron pool.

### Statistics

Behavioral data in the reaching/grasping task were subjected to statistical analysis using one-way repeated measures ANOVA with Bonferroni test. Anatomical data from anterograde labeling of CST fibers were subjected to statistical analysis using one-way ANOVA with Dunnett test. All data were represented as mean ± SEM and statistically accepted at *P* < 0.05.

## Additional Information

**How to cite this article**: Nakagawa, H. *et al*. Reorganization of corticospinal tract fibers after spinal cord injury in adult macaques. *Sci. Rep*. **5**, 11986; doi: 10.1038/srep11986 (2015).
